# Thermodynamic Explanation of Landau Damping by Reduction to Hydrodynamics

**DOI:** 10.3390/e20060457

**Published:** 2018-06-12

**Authors:** Michal Pavelka, Václav Klika, Miroslav Grmela

**Affiliations:** 1 Mathematical Institute, Faculty of Mathematics and Physics, Charles University, Sokolovská 83, 186 75 Prague, Czech Republic; 2 Department of Mathematics, FNSPE, Czech Technical University in Prague, Trojanova 13, 120 00 Prague, Czech Republic; 3 École Polytechnique de Montréal, C.P.6079 suc. Centre-ville, Montréal, QC H3C 3A7, Canada

**Keywords:** Landau damping, entropy, non-equilibrium thermodynamics, Ehrenfest reduction

## Abstract

Landau damping is the tendency of solutions to the Vlasov equation towards spatially homogeneous distribution functions. The distribution functions, however, approach the spatially homogeneous manifold only weakly, and Boltzmann entropy is not changed by the Vlasov equation. On the other hand, density and kinetic energy density, which are integrals of the distribution function, approach spatially homogeneous states strongly, which is accompanied by growth of the hydrodynamic entropy. Such a behavior can be seen when the Vlasov equation is reduced to the evolution equations for density and kinetic energy density by means of the Ehrenfest reduction.

## 1. Introduction

Dynamics of self-interacting gas is well described by the Vlasov equation,

(1) $$\frac{\partial f(t,r,p)}{\partial t}=-\frac{p}{m}\cdot \frac{\partial f(t,r,p)}{\partial r}-F\left(f\left(t,r,p\right)\right)\cdot \frac{\partial f}{\partial p},$$

where *m* is the mass of one particle and f(t,r,p) is the one-particle distribution function on phase space and F is the force exerted on the gas from outside or by the gas itself. The force can be derived from a Hamiltonian (or energy of the system) as follows. For simplicity of notation, the dependence of the distribution function on time or on position and momentum will not always be written explicitly.

Energy of the system with a self-consistent long-range interaction is

(2) $$E=\int dr\int dp\frac{p^{2}}{2m}f+\frac{1}{2}\int dr\int dp\int dr^{\prime }\int dp^{\prime }f\left(r,p\right)V(|r-r^{\prime }\left|\right)f\left(r^{\prime },p^{\prime }\right),$$

where $V(|r-r^{\prime }|)$ is an interaction potential, e.g., electrostatic or gravitational potential. This is the mean-field approximation of the electric energy of the plasma, which is sufficient for discussing the Landau damping below. Derivative of energy with respect to the distribution functions is then

(3) $$\frac{\delta E}{\delta f(r,p)}=\frac{p^{2}}{2m}+\int dr^{\prime }\int dp^{\prime }V(|r-r^{\prime }\left|\right)f\left(r^{\prime },p^{\prime }\right).$$

The force is then given by

(4) $$F\left(t,r\right)=-\frac{\partial }{\partial r}\frac{\delta E}{\delta f}=-\int dr^{\prime }\int dp^{\prime }\frac{\partial V(|r-r^{\prime }|)}{\partial r}f\left(r^{\prime },p^{\prime }\right).$$

Note that this dependence of the force on the distribution function makes the Vlasov equation nonlinear. All terms in the Vlasov Equation (1) have now been specified.

In plasma physics, Landau damping is usually shown for the Vlasov equation coupled with the Poisson equation for electrostatic potential. In three dimensions, the fundamental solution of Poisson equations (Green’s function) is proportional to the Coulomb potential, which means that one can write down the solution to the Poisson equation and plug it back into the Vlasov equation. This way, the mean-field approximation is obtained. However, the situation is more complex for periodic domains as discussed in [1], but the mean-field approximation can still be used. Note also that the mean-field Vlasov equation describes also gravitational interaction and was used by Jeans to study gravitational collapse, which occurs on distances larger than the Jeans length (see [2]).

Landau damping is a feature of solutions of the Vlasov equation—solutions approach spatially homogeneous distributions. This was first derived by L. D. Landau [3,4] when considering the linearized Vlasov equation. Villani and Mouhot then proved it without the linearization, i.e., in the fully nonlinear setting (see [1,5], where conditions on the initial perturbation and decay rates can be found). More precisely, Landau damping can be seen as a development of fast oscillation in the p-dependence of f(t,r,p) and a simultaneous decay of the dependence on r. The distribution function approaches a spatially homogeneous, i.e., r-independent, distribution function only weakly (i.e., not pointwise but in the mean) due to the fast oscillations in the p-dependence. However, the approach of integral over the p-coordinate (particle density ρ(r)) is already strong, i.e.,

(5) $$f\left(r,p\right)\overset{w}{\to }\tilde{f}\left(p\right)\;\mathrm{and}\;\rho \left(r\right)=\int dpmf\left(r,p\right)\to \tilde{\rho }=\mathrm{const}.$$

Constant *m* is the mass of one particle. These two properties of solutions to the Vlasov equation are referred to as the Landau damping.

A physical explanation of Landau damping was given for instance in [6], where particles “surfing” on the electromagnetic waves either give energy to the wave or take energy from it (damping). Landau damping can be also studied by means of statistical mechanics via observing coarse-grained entropies [7,8]. It can be also observed in continuum simulations of the Vlasov equation and electromagnetic field, e.g., [9]. The emergence of dissipative phenomena from reversible equations is also observed when proposing closure relations in the Grad hierarchy, e.g., [10,11,12,13].

Villani [5] summarizes the results of the analytic computations in the linear (the second term on the R.H.S. of Equation (1) is linearized). Landau damping as follows. Under certain assumptions (the data are analytic and the Penrose stability condition holds), solutions to the linearized Vlasov equation have the following properties: (i) all nonzero spatial Fourier modes of $\rho^{1}\left(t,r\right)=\int h\left(t,r,p\right)dp$ (*h* stands for the perturbation of a spatially homogeneous $f_{0}$ distribution function), converge to 0, hence $\rho^{1}$ converges to a constant in space; (ii) the force also converges to 0; (iii) ∫h(t,r,p)φ(r,p)drdp converges to $\int h_{i}\left(r^{\prime },p\right)\varphi \left(r,p\right)drdr^{\prime }dp$ for any smooth test function φ. Here, $h_{i}\left(r,p\right)=h\left(0,r,p\right)$ stands for the initial value of the perturbation, and the extra integration comes from the convergence to the mean.

The nonlinear Landau damping enjoys the same long-time properties if the initial perturbation is a perturbation of a stable equilibrium and some other assumptions are satisfied—particularly that the interaction potential be no more singular than Newtonian (Fourier transpose $\hat{W}\left(k\right)={O(1/|k|}^{2})$ for $k\in Z^{d}$). The main two observations are again that (i) $f\left(t,r,p\right)\to f_{+\infty }\left(p\right)$ weakly, which implies convergence of smooth observables, that is $\int f\left(t,r,p\right)\varphi \left(r,p\right)drdp\to \int f_{+\infty }\left(p\right)\varphi \left(r^{\prime },p\right)drdr^{\prime }dp=V\int f_{+\infty }\left(p\right)\varphi \left(r^{\prime },p\right)dr^{\prime }dp$), and F(t,r)→0 strongly with time.

Landau damping is also interesting from the thermodynamic point of view. Although it clearly described decay of the distribution function to homogeneous equilibria, the Boltzmann entropy (see e.g., [14]),

(6) $$S^{\left(B\right)}\left(f\right)=-k_{B}\int dr\int dpf\left(r,p\right)\left(\ln\left(h^{3}f\left(r,p\right)\right)-1\right),$$

is kept constant by the Vlasov equation, as can be verified by direct calculation. Actually, any functional of the form ∫dr∫dpη(f), η being a function from real numbers to real numbers, is conserved for any energy. Such functionals are called Casimirs of the Poisson bracket. This seems to be in contradiction with the usual thermodynamic interpretation of relaxation to some equilibrium, which is usually associated with growth of entropy. This seemingly paradoxical situation will be resolved in this paper by showing that the hydrodynamic entropy, which is more macroscopic than the Boltzmann entropy, grows while Boltzmann entropy is conserved, which seems to be in agreement with the the answer of Villani [15] when we asked him.

In their rigorous analysis of solutions to the Vlasov kinetic equation, Villani et al. have observed that the emergence of irregularities of solutions plays an analogical role in the kinetic theory as the emergence of ergodicity of particle trajectories in the particle dynamics (connected to the KAM theorem). This observation supports our analysis of solutions of the Vlasov kinetic equation that uses the methods of statistical mechanics. All methods of statistical mechanics (including in particular the Zwanzig-Mori approach [16,17]) are devised to extract important features and ignore the unimportant details. The important features emerge as patterns in the collection of trajectories (i.e., solutions) of the time evolution equations. The longer the time period for which we know the trajectories, the larger is the chance that we recognize the pattern. For this reason, we turn in this paper to the Ehrenfest method that follows the trajectories beyond the infinitesimal period shown in the vector field.

In this paper, we follow [18] (In [18], we investigate Landau damping from a more detailed level of description. In particular, extended kinetic theory with induced dissipation is shown to reproduce Landau damping. In the present work, however, we analyze Landau damping from a less detailed (hydrodynamic) level of description) and investigate the Vlasov Equation (1) by applying methods of statistical mechanics. This investigation offers us a possibility to see both the statistical methods and the Landau damping in a new perspective. In [18], we have first extended the Vlasov equation to include explicitly the micro-turbulence and then we have shown that its decay brings about the Landau damping. In this paper, we apply the methods of non-equilibrium statistical mechanics, namely the Maximum Entropy Principle (MaxEnt) and the Ehrenfest reduction method (see more in Section 2) directly to the Vlasov equation. We reduce it to two equations governing the time evolution of two scalar fields, ρ(r)=∫dpmf(r,p) and $\epsilon \left(r\right)=\int dp\frac{p^{2}}{2m}f\left(r,p\right)$. From an analysis of their solutions, we are then able to see the Landau damping as the approach of the field ρ(r) to a spatially homogeneous field (in Section 3).

## 2. Ehrenfest Reduction

A reduction of a dynamical system (DS1) to another dynamical system (DS2) is a pattern recognition in the phase portrait corresponding to DS1. The phase portrait corresponding to DS1 is a collection of the trajectories that arise in DS1 (i.e., solutions to the governing equations of DS1). The recognized pattern in the DS1 phase portrait is the phase portrait corresponding to DS2. The first step in the reduction is thus an information about the phase portrait. How shall we obtain such information? We recall two examples.

First, it is the Gibbs reduction of the Liouville equation to the equilibrium thermodynamics. The phase portrait is assumed to be ergodic and the recognized pattern is the Gibbs equilibrium distribution function obtained by maximizing the Gibbs entropy with constraints (MaxEnt principle). In the Gibbs analysis, the constraints are the state variables of the equilibrium thermodynamics (i.e., the total mass and the total energy). The Gibbs entropy is a measure of disorder. Since the constraints remain constant during the time evolution, the reduced time evolution is no time evolution.

The second example is the Ehrenfest reduction of DS1 to DS2. The constraints are now fields that serve as state variables in DS2. These two fields do not remain constant in the DS1 time evolution. The pattern is again obtained by the MaxEnt principle and its time evolution by following the DS1 time evolution in a small time interval. Below, we describe the Ehrenfest reduction in detail (in the rest of this section) and then we apply it on the Vlasov Equation (1) in Section 3.

### 2.1. General Formulation

Let us first recall the Ehrenfest reduction, which was developed in [19,20]. The starting point is to expand solutions to the DS1 time evolution equation (denoted by state variables x),

(7) $$\dot{x}=J\left(x\right)$$

in Taylor series in time (i.e., close to the initial condition ${x\left(t+\tau \right)|}_{\tau =0}=x\left(t\right)$),

(8) $$x\left(t+\tau \right)=x\left(t\right)+\tau \dot{x}{{|}_{t}+\frac{\tau^{2}}{2}\ddot{x}|}_{t}+O\left(\tau^{3}\right).$$

By the symbol x, we denote the state variables of the DS1 dynamics. Substituting Equation (7) into the expansion leads to

(9) $$x\left(t+\tau \right)=x\left(t\right)+\tau J\left(x\left(t\right)\right)+\frac{\tau^{2}}{2}\langle \frac{\delta J(x(t))}{\delta x},J\left(x\left(t\right)\right)\rangle +O\left(\tau^{3}\right),$$

where 〈•,•〉 stands for the usual $L^{2}$-product, i.e., integration over the respective space or phase-space domain.

A less detailed level of description, i.e., DS2, has state variables

(10) y=〈π,x〉,

where 〈π,•〉 is the projection operator, derivatives of which $\frac{\delta \pi^{a}}{\delta x^{j}}$ will be denoted simply as $\pi_{i}^{a}$. For linear constant-in-time projection operators, the derivatives constitute a constant matrix, and the angular brackets have the same meaning, i.e., scalar product, as above.

Exact evolution of variables y can be obtained by

(11) $$\dot{y}=\langle \pi ,\dot{x}\rangle =\langle \pi ,J\left(x\right)\rangle ,$$

but, in order to evaluate it, it would be necessary to solve Equation (7). Since the aim is to obtain evolution equation for y only in terms of y, a reduction has to be carried out. In other words, some information has to be forgotten. The approximation can be sought by following two routes: (i) projecting the more detailed evolution to the less detailed state variables; (ii) expanding the sought evolution equation on the coarser level. Comparison of these two approaches yields an assessment of Taylor series expansion of the sought evolution equation on the coarser level (to arbitrary order).

In the latter route, we search for the unknown operator ϕ that governs the evolution on the coarser level DS2

$$\dot{y}=\phi \left(y\right).$$

Expanding its solution around an arbitrary initial condition y(t) yields

(12) $$y_{k}\left(t+\tau \right)=y_{k}\left(t\right)+\tau \phi_{k}\left(y\left(t\right)\right)+\frac{\tau^{2}}{2}\frac{\delta \phi_{k}}{\delta y_{j}}\phi_{j}.$$

To identify the asymptotic expansion of the unknown operator ϕ, we write

(13) $$\phi_{k}=R_{k}^{\left(0\right)}+\tau R_{k}^{\left(1\right)}+\frac{\tau^{2}}{2}R_{k}^{\left(2\right)}+O\left(\tau^{3}\right).$$

Now, we compare this coarser DS2 level expansion to the DS1 time evolution. In order to achieve that, however, the DS1 time evolution, has to be made dependent on y. We choose the mapping from y to x to be the MaxEnt mapping $\tilde{x}\left(y\right)$, i.e., to find x such that the detailed entropy S(x) is maximal subject to the constraints represented by y=π(x). Then, by substituting (13) into (12)) with projection of Equation (9), we obtain

(14) $$\langle \pi ,x\left(t+\tau \right)\rangle =\langle \pi ,x\left(t\right)\rangle +\tau \langle \pi ,J\left(\tilde{x}\left(y\right)\right)\rangle +\frac{\tau^{2}}{2}\langle \pi ,\langle \frac{\delta J(x(t))}{\delta x}{|}_{\tilde{x}\left(y\right)},J\left(\tilde{x}\left(y\right)\right)\rangle \rangle +O\left(\tau^{3}\right),$$

where the quasi-equilibrium $\tilde{x}\left(y\right)$ initial conditions were chosen so that only y appears in the expansion. The comparison leads to

(15) $$\begin{array}{cc} y_{k} & =\langle \pi_{k},\tilde{x}\left(y\right)\rangle \;\ldots \;\mathrm{from}\;\tau^{0}\;\mathrm{coefficient}, \end{array}$$

(16) $$\begin{array}{cc} R_{k}^{\left(0\right)} & =\langle \pi_{k},J\left(\tilde{x}\left(y\right)\right)\rangle \;\ldots \;\mathrm{from}\;\tau^{1}, \end{array}$$

and

(17) $$\begin{array}{cc} R_{k}^{\left(1\right)} & =\frac{1}{2}\left(\langle \pi_{k},\langle \frac{\delta J(x(t))}{\delta x}{|}_{\tilde{x}\left(y\right)},J\left(\tilde{x}\left(y\right)\right)\rangle \rangle -\langle \frac{\delta R_{k}^{\left(0\right)}}{\delta y_{j}},R_{j}^{\left(0\right)}\rangle \right)\;\ldots \;\mathrm{from}\;\tau^{2}\\ & =\frac{1}{2}\left(\langle \pi_{k},\langle \frac{\delta J(x(t))}{\delta x}{|}_{\tilde{x}\left(y\right)},J\left(\tilde{x}\left(y\right)\right)\rangle \rangle -\langle \frac{\delta \langle \pi_{k},J\left(\tilde{x}\left(y\right)\right)\rangle }{\delta y_{j}},\langle \pi_{j},J\left(\tilde{x}\left(y\right)\right)\rangle \rangle \right), \end{array}$$

which completes the estimate of the sought evolution equation on the coarser level via (13)

(18) $${\dot{y}}_{k}=\phi_{k}\left(y\right)\approx R_{k}^{\left(0\right)}+\tau R_{k}^{\left(1\right)}+O\left(\tau^{2}\right).$$

Note that the $\tau^{0}$ term has to be strictly satisfied due to the requirement that both the more and less detailed evolution coincide at τ=0 on the quasi-equilibrium. The zeroth correction $R_{k}^{\left(0\right)}$ can be also naturally understood as the least biased way to express x in terms of y is the MaxEnt [21] mapping from y to x, $\tilde{x}\left(y\right)$, which is exactly what the first approximation is. The first correction $R_{k}^{\left(1\right)}$ is typically non-zero as the detailed evolution (7) carries x from the quasi-equilibrium $\tilde{x}\left(y\right)$ to values that are not in the image of the MaxEnt mapping, i.e., out of the quasi-equilibrium (or Legendre) submanifold, and corrections have to be introduced.

### 2.2. Hamiltonian Version of Ehrenfest Reduction

The purpose of this section is to reformulate the Ehrenfest reduction in the case when the detailed evolution DS1 is Hamiltonian.

#### 2.2.1. Hamiltonian Structure of the Vlasov Equation

Vlasov Equation (1) is a Hamiltonian evolution, since it is generated by the Boltzmann- Poisson bracket

(19) $${\left\{A,B\right\}}^{\left(B\right)}=\int dr\int dpf\left(\frac{\partial }{\partial r}\frac{\delta A}{\delta f}\frac{\partial }{\partial p}\frac{\delta B}{\delta f}-\frac{\partial }{\partial r}\frac{\delta B}{\delta f}\frac{\partial }{\partial p}\frac{\delta A}{\delta f}\right),$$

which is of course antisymmetric and fulfills both the Leibniz rule and Jacobi identity. The Hamiltonian structure of plasma evolution equations was first found by Phil Morrison and John Greene in [22]. See also [23] or [24]. By rewriting the bracket as

(20) $$\int dr\int dp\frac{\delta A}{\delta f}\cdot \frac{\partial f}{\partial t}={\left\{A,E\right\}}^{\left(B\right)}=\int dr\int dp\frac{\delta A}{\delta f}\cdot \mathrm{rhs},$$

the terms rhs are the right-hand side of the evolution equation for *f*, which is the Vlasov Equation (1). This Poisson bracket can be derived from the Liouville Poisson bracket by projection or it can be seen as the Lie-Poisson bracket corresponding to symplectic transformations on the one-particle cotangent bundle (see, e.g., [25]).

Any real-valued function of the distribution function σ:R→R then generates a Casimir of the bracket,

(21) $${\left\{A,\int dr\int dp\sigma \left(f\right)\right\}}^{\left(B\right)}=0\;\forall A\left(f\right).$$

This means in particular that

(22) $${\dot{S}}^{\left(B\right)}=\left\{S^{\left(B\right)},E\right\}=0,$$

and Boltzmann entropy is thus indeed conserved by the Vlasov equation. The Boltzmann entropy is a plausible entropy (a Casimir of the Boltzmann Poisson bracket), but it is not the only one possible. Nevertheless, in the case of ideal gases, it follows by MaxEnt from the Liouville entropy (see e.g., [14]), which can be seen as the phase-space analogue of the Shannon entropy $-k_{B}\sum_{i}p_{i}\ln p_{i}$. Shannon entropy is uniquely determined by Shannon’s axioms defining uncertainty [26]. In this sense, the Boltzmann entropy $S^{\left(B\right)}$ is unique. Moreover, energy is conserved automatically due to the antisymmetry of the Poisson bracket.

Each Poisson bracket can be seen as constructed by means of a Poisson bivector L,

(23) $$\left\{A,B\right\}=A_{x^{i}}L^{ij}B_{x^{j}}.$$

Antisymmetry of the Poisson bracket is expressed by antisymmetry of the Poisson bivector, and Jacobi identity for the bracket is also inherited from a corresponding formula for the bivector (see e.g., [27] p. 332).

Casimirs of the Poisson bracket are functionals that are not evolving regardless of the choice of energy, i.e.,

(24) $$\dot{S}=\left\{S,E\right\}=0\;\forall E,$$

which means that

(25) $$S_{x^{i}}L^{ij}=0\;\forall j.$$

This relation is satisfied for all Casimirs. In particular, entropy is always taken as a Casimir of the Poisson bracket in order to be conserved by the Hamiltonian evolution, which means that the above relation holds for *S* being entropy. More generally, this allows a clear separation of reversible and irreversible part of evolution dynamics.

#### 2.2.2. Formal Solution of Hamiltonian Evolution

The first step in Ehrenfest reduction was to expand the evolution of detailed variables x as a Taylor series in time. Assume that evolution of x is purely Hamiltonian, which means that

(26) $${\dot{x}}^{i}=\underset{=J^{i}}{\underset{︸}{L^{ij}E_{x^{j}}}}\;\mathrm{or}\;\dot{A}\left(x\right)=\left\{A,E\right\}\;\forall A.$$

Solution to the Hamiltonian evolution equations can be expanded in series as (see e.g., [27] pp. 334)

(27) $$A\left(x\left(t+\tau \right)\right)=A\left(x\left(t\right)\right)+\tau \left\{A,E\right\}+\frac{\tau^{2}}{2}\left\{\left\{A,E\right\},E\right\}+O\left(\tau^{3}\right).$$

When expanding the Poisson brackets by means of the Poisson bivector and when taking into account only linear functionals *A*, i.e., $A\left(x\right)=A_{i}x^{i}$, this last equation becomes

(28) $$A_{i}x^{i}\left(t+\tau \right)=A_{i}x^{i}\left(t\right)+\tau A_{i}L^{ij}E_{x^{j}}+\frac{\tau^{2}}{2}A_{i}\frac{\partial }{\partial x^{k}}\left(L^{ij}E_{x^{j}}\right)L^{kl}E_{x^{l}}+O\left(\tau^{3}\right)\;\forall A_{i},$$

which is the analogue of Equation (9).

Taylor expansion (28) can be interpreted as a new evolution equation for $x^{i}$

(29) $$\frac{dx^{i}}{d\tau }=L^{ij}E_{x^{j}}+\frac{\tau }{2}\frac{\partial }{\partial x^{k}}\left(L^{ij}E_{x^{j}}\right)L^{kl}E_{x^{l}},$$

where the first term on the right-hand side generates reversible evolution while the second irreversible. Such an evolution equation can be regarded as a *self-regularized* evolution of $x^{i}$ corresponding to time step τ. A similar idea has already been brought up in symplectic integration [28].

The original Hamiltonian evolution, Equation (26), conserved both energy (due to antisymmetry) and entropy (due to degeneracy of the Poisson bracket). None of these properties is kept in the self-regularized evolution Equation (29). Indeed, evolution of entropy becomes

(30) $$\begin{array}{ccc} \frac{dS}{d\tau } & = & S_{x^{i}}\frac{dx^{i}}{d\tau }=\underset{=0}{\underset{︸}{S_{x^{i}}L^{ij}}}E_{x^{j}}+\frac{\tau }{2}S_{x^{i}}\frac{\partial }{\partial x^{k}}\left(L^{ij}E_{x^{j}}\right)L^{kl}E_{x^{l}}\\ & = & \frac{\tau }{2}\frac{\partial }{\partial x^{k}}\left(\underset{=0}{\underset{︸}{S_{x^{i}}L^{ij}}}E_{x^{j}}\right)L^{kl}E_{x^{l}}-\frac{\tau }{2}S_{x^{k}x^{i}}L^{ij}E_{x^{j}}L^{kl}E_{x^{l}}\\ & = & \frac{\tau }{2}L^{ij}E_{x^{j}}\left(-S_{x^{i}x^{k}}\right)L^{kl}E_{x^{l}}\geq 0. \end{array}$$

The inequality follows from concavity of S(x) (and thus negative semi-definiteness of $d^{2}S$). Entropy is thus produced by the self-regularized equations. Similarly, evolution of energy becomes

(31) $$\frac{dE}{d\tau }=E_{x^{i}}\frac{dx^{i}}{d\tau }=-\frac{\tau }{2}L^{ij}E_{x^{j}}E_{x^{i}x^{k}}L^{kl}E_{x^{l}}\leq 0$$

due to convexity of energy ($d^{2}E$ positive semi-definite). Energy is thus dissipated by the self-regularized evolution.

#### 2.2.3. Projection of the Poisson Bracket

Assume now that when functionals dependent only on the less detailed variables y are plugged into the Poisson bracket, the resulting expression depends only on variables y and it is referred to as the less-detailed Poisson bracket ${}^{↓}\left\{•,•\right\}$, i.e.,

(32) $$\left\{A\left(y\right),B\left(y\right)\right\}=A_{y^{a}}\underset{={{}^{↓}L}^{ab}\left(y\right)}{\underset{︸}{\pi_{i}^{a}L^{ij}\pi_{j}^{b}}}B_{y^{b}}{=}^{↓}\left\{A,B\right\}.$$

That is the case for example when y are the hydrodynamic fields of density, momentum density and entropy density and x the one-particle distribution functions. Poisson brackets can often be derived from more detailed Poisson brackets by such projections (see, e.g., [25]).

The projections are naturally made in the so-called energetic representation (see [29]), where, for example, in the case of hydrodynamic field entropy density is among the state variables instead of energy density. On the other hand, in the entropic representation, the field of energy density is among the state variables. In that case, it is natural to employ the MaxEnt principle to find the least biased estimate of the detailed variable x based on the knowledge of the less detailed variables y. In the case of the energetic representation, the MaxEnt principle is replaced by the principle of minimal energy: find x such that energy E(x) is minimal subject to the knowledge of field y that include the less detailed entropy density. This can be expressed by Legendre transformation

(33) $$\frac{\partial }{\partial x}\left(-E\left(x\right)+y^{*}\cdot y\left(x\right)\right){|}_{\tilde{x}\left(y\right)}=0,$$

which gives the dependence $\tilde{x}\left(y^{*}\right)$ and a new functional

(34) $${}^{↓}E^{*}\left(y^{*}\right)=-E\left(\tilde{x}\left(y\right)\right)+y^{*}\cdot y\left(\tilde{x}\left(y^{*}\right)\right),$$

the conjugate lower energy, which is a functional of conjugate less detailed (lower) variables $y^{*}$. The energy on the lower level is then obtained by a subsequent Legendre transformation

(35) $$\frac{\partial }{\partial y^{*}}\left(-{}^{↓}E^{*}\left(y^{*}\right)+y^{*}\cdot y\right){|}_{y^{*}\left(y\right)}=0$$

and

(36) $${}^{↓}E\left(y\right)=-{}^{↓}E^{*}\left(y^{*}\left(y\right)\right)+y^{*}\left(y\right)\cdot y.$$

These equations imply, in particular, that

(37) $${}^{↓}E_{y_{a}^{*}}^{*}=y^{a}\;\mathrm{and}\;{}^{↓}E_{y^{a}}=y_{a}^{*}.$$

From Equation (33), it then follows that

(38) $$E_{x^{i}}{|}_{\tilde{x}\left(y\right)}=y_{a}^{*}\frac{\partial \pi^{a}}{\partial x^{i}}=\frac{\partial {}^{↓}E}{\partial y^{a}}\frac{\partial \pi^{a}}{\partial x^{i}},$$

which will be useful later.

Assuming that we have already projected the Poisson bracket to the lower level of description, evolution of the less detailed state variables is given by

(39) $${\dot{y}}^{a}=\underset{={{}^{↓}J}^{a}}{\underset{︸}{{{}^{↓}L}^{ab}{}^{↓}E_{y^{b}}}}\;\mathrm{or}\;\dot{A}{=}^{↓}\left\{A,{}^{↓}E\right\}\forall A\left(y\right).$$

The Taylor expansion in time on the less-detailed level of description then yields

(40) $$A_{a}y^{a}\left(t+\tau \right)=A_{a}y^{a}\left(t\right)+\tau A_{a}{{}^{↓}L}^{ab}{}^{↓}E_{y^{b}}+\frac{\tau^{2}}{2}A_{a}\frac{\partial }{\partial y^{c}}\left({{}^{↓}L}^{ab}{}^{↓}E_{y^{b}}\right){{}^{↓}L}^{cd}{}^{↓}E_{y^{d}}+O\left(\tau^{3}\right)\;\forall A_{a}.$$

This last equation is the analogue of Equation (12), and can be regarded as evolution equation

(41) $$\frac{dy^{a}}{d\tau }={{}^{↓}L}^{ab}{}^{↓}E_{y^{b}}+\frac{\tau }{2}\frac{\partial }{\partial y^{c}}\left({{}^{↓}L}^{ab}{}^{↓}E_{y^{b}}\right){{}^{↓}L}^{cd}{}^{↓}E_{y^{d}},$$

where the right-hand side consists of a reversible (first) and an irreversible (second) term. This is again a *self-regularization* of evolution (39).

#### 2.2.4. Comparing the Solutions on Different Levels

The next step in the Ehrenfest reduction is to alter the right-hand side of the less-detailed evolution equation so that the solution is closer to the solution on the detailed level of description.

**Figure 1:**
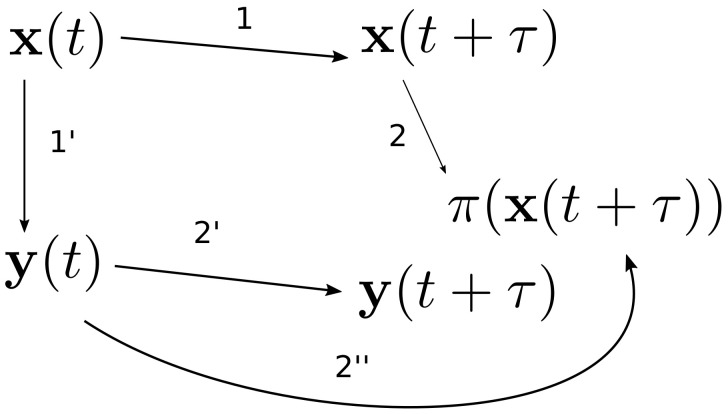
Hamiltonian interpretation of the Ehrenfest reduction. Step 1: The exact more detailed evolution equations are first solved formally to obtain their solution at time t+τ. Step 2: This solution, x(t+τ), is then projected to the less detailed level to obtain π(x(t+τ)). Step $1^{\prime }$: Alternative route is to first project x(t) to y(t). Step $2^{\prime }$: The less detailed evolution equation (generated by the projection of the Poisson bracket) then takes y(t) and gives y(t+τ). Step $2^{\prime \prime }$: We have thus π(x(t+τ)) and y(t+τ), which should ideally be equal, but they are typically not. The value π(x(t+τ)) is of course more precise because it is constructed from the detailed evolution equations. To make the value y(t+τ) more precise, the less detailed evolution equations are altered by adding the difference between the self-regularized detailed and less-detailed equations. Such equations for y are then the reduced Ehrenfest equations.

More precisely, Taylor expansion (28) gives an approximation of $x^{i}\left(t+\tau \right)$ provided $x^{i}\left(t\right)$ is known. Similarly, Taylor expansion (40) gives an approximation of $y^{a}\left(t+\tau \right)$ provided $y^{a}\left(t\right)$ is known. The value $y^{a}\left(t+\tau \right)$ can be, however, approached by two ways: (i) Projection from x(t) to y(t) and subsequent evolution by Taylor expansion (40) or (ii) evolution by expansion (28) and subsequent projection from x(t+τ) to y(t+τ). The second route is of course more precise, but one has to solve the detailed evolution equations for x. Therefore, the first route is more suitable, but it needs a further correction to make it closer to the second route. An additional term must thus be added to expansion (40), which makes the two routes equivalent up to the given order of τ. This procedure is summarized in Figure 1.

Let us thus compare the self-regularized evolutions (28) and (40). The first terms of the expansions are already related by the projection because (using Equation (38))

(42) $$\pi_{i}^{a}L^{ij}E_{x^{j}}{|}_{\tilde{x}\left(y\right)}=\pi_{i}^{a}L^{ij}\pi_{j}^{b}{}^{↓}E_{y^{b}}={{}^{↓}L}^{ab}{}^{↓}E_{y^{b}}.$$

The second terms of the expansions are no longer related by such a projection. Therefore, one has to add the difference between these two second terms. The regularized evolution equation for y then becomes

(43) $${\dot{y}}^{a}={{}^{↓}L}^{ab}E_{y^{b}}+\frac{\tau }{2}\left(\pi_{i}^{a}\frac{\partial }{\partial x^{k}}\left(L^{ij}E_{x^{j}}\right)L^{kl}E_{x^{l}}-\frac{\partial }{\partial y^{c}}\left({{}^{↓}L}^{ab}{}^{↓}E_{y^{b}}\right){{}^{↓}L}^{cd}{}^{↓}E_{y^{d}}\right){|}_{\tilde{x}\left(y\right)},$$

which is the less-detailed evolution equation obtained by the Ehrenfest reduction, analogue of Equation (18).

#### 2.2.5. The Special Case of Constant Poisson Bivector

Hamilton canonical equations of classical mechanics are generated by the canonical Poisson bracket or canonical Poisson bivector,

(44) $${\left\{A,B\right\}}^{\left(can\right)}=A_{r}\cdot B_{p}-A_{p}\cdot B_{r},\;L=\left(\begin{array}{cc} 0 & 1\\ -1 & 0 \end{array}\right).$$

The canonical Poisson bivector is a constant matrix.

The Poisson bracket for hydrodynamic density and momentum density (ρ,u) is not a canonical one. However, it can be casted into the canonical form by transformation from (ρ,u) into the so-called Clebsch variables (see [30] and [31], or even (ρ,u,s) as in [32]). Constant Poisson bivectors can be thus often met.

If the bivector is constant, expansions of the solutions (28) and (40) obtain a particularly simple form,

(45) $$\begin{array}{cc} x^{i}\left(t+\tau \right) & =x^{i}\left(t\right)+\tau L^{ij}E_{x^{i}}+\frac{\tau^{2}}{2}\underset{=-M^{ik}}{\underset{︸}{L^{ij}\frac{\delta^{2}E}{\delta x^{j}x^{k}}L^{kl}}}E_{x^{l}}+O\left(\tau^{3}\right), \end{array}$$

(46) $$\begin{array}{cc} y^{a}\left(t+\tau \right) & =y^{a}\left(t\right)+\tau {{}^{↓}L}^{ab}E_{y^{b}}+\frac{\tau^{2}}{2}\underset{=-{{}^{↓}M}^{ad}}{\underset{︸}{{{}^{↓}L}^{ab}\frac{\delta^{2}E}{\delta y^{c}y^{b}}{{}^{↓}L}^{cd}}}E_{y^{d}}+O\left(\tau^{3}\right), \end{array}$$

which gives evolution equations

(47) $$\begin{array}{cc} {\dot{x}}^{i}\left(t\right) & =L^{ij}E_{x^{i}}-\frac{\tau }{2}M^{ij}E_{x^{j}}, \end{array}$$

(48) $$\begin{array}{cc} {\dot{y}}^{a}\left(t\right) & ={{}^{↓}L}^{ab}E_{y^{b}}-\frac{\tau }{2}{{}^{↓}M}^{ab}E_{y^{b}}. \end{array}$$

Matrices M and ${}^{↓}M$ are clearly symmetric. Moreover, it follows from convexity of energy, the second differential of which is thus a positive definite matrix, that

(49) $$v_{i}M^{il}v_{l}=-v_{i}L^{ij}E_{x^{j}x^{k}}L^{kl}v_{l}=L^{ji}v_{i}E_{x^{j}x^{k}}L^{kl}v_{l}\geq 0\;\forall v,$$

and the matrices M and ${}^{↓}M$ are thus symmetric positive semidefinite. In particular, it means that energy is destroyed in both equations (47) and (48). Evolution Equations (47) and (48) can be interpreted as self-regularizations of the original completely reversible evolution Equations (26) and (39).

The Ehrenfest reduced evolution equation is then

(50) $${\dot{y}}^{a}\left(t\right)={{}^{↓}L}^{ab}E_{y^{b}}-\frac{\tau }{2}\left(\pi_{i}^{a}M^{ij}{{|}_{\tilde{x}\left(y\right)}E_{x^{j}}|}_{\tilde{x}\left(y\right)}-{{}^{↓}M}^{ab}E_{y^{b}}\right),$$

which is the analogue of Equation (18).

#### 2.2.6. Canonical Hamiltonian System

For instance, the canonical Poisson bivector (44) is constant. The canonical Poisson bracket, which provides natural kinematics on cotangent bundles, is formed from the Poisson bivector.

Let the variables on a cotangent bundle be denoted by (r,p), which can be interpreted as position and momentum of a particle. Evolution of these variables is then given by

(51) $$\left(\begin{array}{c} \dot{r}\\ \dot{p} \end{array}\right)=\left(\begin{array}{cc} 0 & 1\\ -1 & 0 \end{array}\right)\cdot \left(\begin{array}{c} H_{r}\\ H_{p} \end{array}\right),$$

with H(r,p) being energy of the particle in a static external field, e.g.,

(52) $$H\left(r,p\right)=\frac{p^{2}}{2m}+V\left(r\right).$$

The self-regularized evolution of (r,p) is then, according to Equation (47),

(53) $$\left(\begin{array}{c} \dot{r}\\ \dot{p} \end{array}\right)=\left(\begin{array}{cc} 0 & 1\\ -1 & 0 \end{array}\right)\cdot \left(\begin{array}{c} H_{r}\\ H_{p} \end{array}\right)-\frac{\tau }{2}\underset{=M}{\underset{︸}{\left(\begin{array}{cc} H_{pp} & -H_{pr}\\ -H_{rp} & H_{rr} \end{array}\right)}}\cdot \left(\begin{array}{c} H_{r}\\ H_{p} \end{array}\right).$$

For the particular choice of Hamiltonian (52), the evolution equations become

(54) $$\left(\begin{array}{c} \dot{r}\\ \dot{p} \end{array}\right)=\left(\begin{array}{c} \frac{p}{m}\\ -V_{r} \end{array}\right)-\frac{\tau }{2}\underset{=M}{\underset{︸}{\left(\begin{array}{cc} \frac{1}{m} & 0\\ 0 & V_{rr} \end{array}\right)}}\cdot \left(\begin{array}{c} V_{r}\\ \frac{p}{m} \end{array}\right)=\left(\begin{array}{c} \frac{p}{m}\\ -V_{r} \end{array}\right)-\frac{\tau }{2m}\left(\begin{array}{c} V_{r}\\ V_{rr}\cdot p \end{array}\right).$$

This is the self-regularized evolution of the particle.

Assuming that the external field V(r) is convex and that it has a minimum, the Hamiltonian (or energy) is also convex. Consequently, the dissipative matrix M is positive-semidefinite, and energy is dissipated in course of the evolution as

(55) $$\dot{H}=-\frac{\tau }{2}\left(\frac{1}{m}{\left(V_{r}\right)}^{2}+\frac{p}{m}\cdot V_{rr}\cdot \frac{p}{m}\right)\leq 0,$$

and the evolution stops at the position and momentum satisfying

(56) $$V_{r}=0\;\mathrm{and}\;p=0,$$

which is the equilibrium position of the particle within the field as well as the position of lowest total energy.

Let us now consider a less detailed level of description where only the position r plays the role of state variable. The mapping from (r,p) to (r) is the projection between the two levels of description. The Poisson bivector on the less detailed description is a 1 × 1 matrix, i.e., a number, and it is given by

(57) $$\left(1,0\right)\cdot L\cdot \left(\begin{array}{c} 1\\ 0 \end{array}\right)=0.$$

The less detailed Poisson bivector is identically zero. The less detailed evolution is thus zero as well as the self-regularized less detailed evolution.

However, the less detailed evolution derived by the Ehrenfest reduction, Equation (50), is not zero and reads

(58) $$\dot{r}=-\frac{\tau }{2m}\left(1,0\right)\cdot \left(\begin{array}{c} V_{r}\\ V_{rr}\cdot \tilde{p}\left(r\right) \end{array}\right)=-\frac{\tau }{2m}V_{r}.$$

This reduced evolution tends again to the minimum of the external field.

Lower-level energy ${}^{↓}E\left(r\right)=H\left(r,\tilde{p}\left(r\right)\right)=V\left(r\right)$ is being damped by the reduced evolution equation until the state with minimal energy is reached. In order to restore energy conservation, another state variable (entropy or energy) should be taken into account so that mechanical energy can be transformed into thermal (see e.g., [14]).

## 3. From Vlasov to Mechanical Equilibrium

Now, we are coming to the main point of the paper—the reduction of the Vlasov equation to mechanical equilibrium. Mechanical equilibrium is the level of description where only density and kinetic energy density play the role of state variables. Since those two fields can still evolve in time, mechanical equilibrium is still a generally non-equilibrium description. The reason for the name is that momentum density is already in a quasi-equilibrium state given by the acting mechanical forces.

The reduced equations will naturally exhibit the tendency to spatially homogeneous equilibrium, which can be interpreted as a manifestation of Landau damping. However, let us first define the projection from the Vlasov level of description to the level of mechanical equilibrium.

### 3.1. Projection

Since the particle density approaches the equilibrium in a strong sense, we can expect that if entropy were a function of the density, it could approach its maximum. Therefore, it is sensible to regard the Vlasov equation from a less detailed (or lower) level of description—for example, the level of mechanical equilibrium where state variables are

(59) $$\begin{array}{cc} \text{particle density}: & \rho \left(r\right)=\int dpmf\left(r,p\right), \end{array}$$

(60) $$\begin{array}{cc} \text{kinetic energy density}: & \varepsilon \left(r\right)=\int dp\frac{p^{2}}{2m}f\left(r,p\right). \end{array}$$

The level of description where ρ and ε play the role of state variables is referred to as the level of mechanical equilibrium (motivated by [33]) because momentum is not present among the state variables.

Let us justify the reduction to the level of mechanical equilibrium from the perspective of rigorous mathematical results summarized in Section 1. Consider a particular choice of the test function that is a tensor product $\varphi \left(r,p\right)=p⊗\tilde{\varphi }\left(r\right)$, where $\tilde{\varphi }$ is smooth. Then, we have that

$$\int h\left(t,r,p\right)\varphi \left(r,p\right)drdp=\int dr\tilde{\varphi }\left(r\right)\int dp\;ph\left(t,r,p\right)\to \int dr\tilde{\varphi }\left(r\right)\int dr^{\prime }dp\;ph_{i}\left(r^{\prime },p\right),$$

where the extra integration with respect to $r^{\prime }$ gives the average towards which the perturbation converges. As this relation holds for all smooth $\tilde{\varphi }$ and particularly for smooth cutoff functions (convolution of a characteristic function with a mollifier), we can observe that

$$\lim_{t\to +\infty }\int dv\;vh\left(t,x,v\right)=\int dydv\;vh_{i}\left(y,v\right),$$

which is a constant in space. Therefore, not only the macroscopic density, but also the macroscopic momentum,

(61) u(t,r)=∫dppf(t,r,p),

tends, as t→∞, to a spatially homogeneous state. Hence, it is natural to consider the mechanical equilibrium as a reasonable level of description for long time behavior of the system. We shall explore this Landau damping via pertinent Ehrenfest reduction that is able to reveal irreversible behavior on the reduced level of description.

Total energy (2) can be expressed as

(62) $$E=\int dr\varepsilon \left(r\right)+\frac{1}{2}\int dr\int dr^{\prime }\frac{\rho (r)}{m}V(|r-r^{\prime }\left|\right)\frac{\rho (r^{\prime })}{m}.$$

Mass and kinetic energy density are thus obtained from the distribution function by projection (59) and (60).

On the other hand, knowledge of fields ρ and ε is insufficient for exact reconstruction of the distribution function. What is the estimate of the distribution function when only those two fields are accessible? The answer is the maximum entropy principle (MaxEnt), which reveals that the least biased estimate of the distribution function is the solution to equation

(63) $$\frac{\delta }{\delta f}\left(-S^{\left(B\right)}\left(f\right)+\int dr\rho^{*}\left(r\right)\rho \left(f\right)+\int dr\varepsilon^{*}\left(r\right)\varepsilon \left(f\right)\right)=0,$$

where fields $\rho^{*}$ and $\varepsilon^{*}$ play the role of Lagrange multipliers ensuring constraints (59) and (60), but can be also given geometrical interpretation [14]. Solving this equation leads to

(64) $$\tilde{f}\left(\rho ,\varepsilon ;p\right)=\frac{1}{h^{3}}\exp\left(-\frac{m\rho^{*}\left(\rho ,\varepsilon \right)}{k_{B}}\right)\exp\left(-\frac{\varepsilon^{*}\left(\rho ,\varepsilon \right)}{k_{B}}\frac{p^{2}}{2m}\right),$$

where the $h^{-3}$ prefactor comes ensures the correct units of the distribution function. Using constraints (59) and (60), the Lagrange multipliers can be expressed as functions of ρ and ε, which finally leads to

(65) $$\tilde{f}\left(\rho ,\varepsilon ;p\right)=\frac{\rho }{m^{4}}{\left(\frac{3}{4\pi }\frac{\rho }{\varepsilon }\right)}^{3/2}\exp\left(-\frac{3}{2}\frac{\rho }{m\varepsilon }\frac{p^{2}}{2m}\right),$$

which is the MaxEnt estimate of the distribution function subject to the knowledge of field ρ and ε.

Boltzmann entropy evaluated at the MaxEnt distribution function then becomes the entropy on the level of mechanical equilibrium,

(66) $$S^{\left(ME\right)}\left(\rho ,\varepsilon \right)=-k_{B}\int dr\frac{\rho }{m}\left(-\frac{5}{2}+\frac{3}{2}\ln\left(\frac{3h^{2}}{4\pi m^{2}}\frac{\rho }{\varepsilon }\right)+\ln\frac{\rho }{m}\right),$$

which is in fact the local equilibrium version of the Sackur-Tetrode equation [29].

Having constructed the projection from *f* onto the level of mechanical equilibrium, a mapping from mechanical equilibrium back into the space of distribution functions and the implied mechanical-equilibrium entropy, we have specified the static relations between the two levels of description (Vlasov and mechanical equilibrium). Let us now have a look at dynamical relations between the levels, i.e., how evolution on one level corresponds with evolution on the another level.

### 3.2. Construction of the Reduced Evolution

Let us now apply the Ehrenfest reduction to the passage from the Vlasov level of description onto the level of mechanical equilibrium. State variables are identified as

(67) x=f(r,p)andy=(ρ(r),ε(r)).

The more detailed evolution equation is the Vlasov Equation (1). The sought reduced evolution equations are evolution equations for ρ and ε.

The zeroth approximation is zero, which readily follows from the observation that

(68) $$J\left(\tilde{x}\left(y\right)\right)=-\frac{p}{m}\cdot \frac{\partial \tilde{f}}{\partial r}-F\left(\tilde{f}\right)\left(t,r\right)\cdot \frac{\partial \tilde{f}}{\partial p},$$

where $\tilde{f}\left(\rho ,\varepsilon ;p\right)$ is the quasi-equilibrium state calculated in (65). The zeroth approximation of the macroscopic density then follows from the corresponding projection of the last expression for $J(\tilde{x}\left(y\right)),$ which is

(69) $$R_{\rho }^{\left(0\right)}=\langle \pi_{\rho },J\left(\tilde{x}\left(y\right)\right)\rangle =\int dpm\left[-\frac{p}{m}\cdot \frac{\partial \tilde{f}}{\partial r}-F\left(\tilde{f}\right)\left(t,r\right)\cdot \frac{\partial \tilde{f}}{\partial p}\right].$$

The first term vanishes as it is an odd function in all $p_{k}$ and the second term can be simply integrated to zero as F(t,r) is independent on momentum p. Analogously, one can show that the projection yielding kinetic energy density ε vanishes as well on $J(\tilde{x}\left(y\right))$ and hence the zeroth approximation $R_{\varepsilon }^{\left(0\right)}$ is zero.

The first approximation that corresponds to irreversible evolution can again be calculated from the relations (17) identified above. As the zeroth approximation is zero, i.e., $R^{\left(0\right)}=\langle \pi ,J\left(\tilde{x}\left(y\right)\right)\rangle =0$, only the first terms contribute to the first order correction. Let us start by calculating the variation of δJ(x)/δx

(70) $$\begin{array}{cc} J(f\left(r^{\prime \prime },p^{\prime \prime }\right) & =-\frac{p_{k}^{\prime \prime }}{m}\frac{\partial f}{\partial r_{k}^{\prime \prime }}\left(r^{\prime \prime },p^{\prime \prime }\right)+\frac{\partial f}{\partial p_{k}^{\prime \prime }}\left(r^{\prime \prime },p^{\prime \prime }\right)\int dr^{\prime }\int dp^{\prime }\frac{\partial V(|r^{\prime \prime }-r^{\prime }|)}{\partial r_{k}^{\prime \prime }}f\left(r^{\prime },p^{\prime }\right)=\\ & =\int dr\int dp\;\delta \left(r-r^{\prime \prime }\right)\delta \left(p-p^{\prime \prime }\right)\\ & \;\cdot \left[-\frac{p_{k}}{m}\frac{\partial f(r,p)}{\partial r_{k}}+\frac{\partial f(r,p)}{\partial p_{k}}\int dr^{\prime }\int dp^{\prime }\frac{\partial V(|r-r^{\prime }|)}{\partial r_{k}}f\left(r^{\prime },p^{\prime }\right)\right], \end{array}$$

where $\frac{\partial f(r^{\prime \prime },p^{\prime \prime })}{\partial r_{k}^{\prime \prime }}{|}_{r^{\prime \prime }=r,p^{\prime \prime }=p}=\frac{\partial f(r,p)}{\partial r_{k}}$ was used. Hence, the variation of δJ(x)/δx is

$$\begin{array}{c} \frac{\delta J(f\left(r^{\prime \prime },p^{\prime \prime }\right))}{\delta f(r,p)}=\partial_{r_{k}}\left(\delta \left(r-r^{\prime \prime }\right)\right)\delta \left(p-p^{\prime \prime }\right)\frac{p_{k}}{m}-\delta \left(r-r^{\prime \prime }\right)\left(\partial_{p_{k}}\delta \left(p-p^{\prime \prime }\right)\right)\\ \;\cdot \int dr^{\prime }\int dp^{\prime }\frac{\partial V(|r-r^{\prime }|)}{\partial r_{k}}f\left(r^{\prime },p^{\prime }\right)+\\ \;+\underset{=\frac{\partial f(r^{\prime \prime },p^{\prime \prime })}{\partial p_{k}^{\prime \prime }}\frac{\partial V(|r^{\prime \prime }-r|)}{\partial r_{k}^{\prime \prime }}}{\underset{︸}{\int dr^{\prime }\int dp^{\prime }\delta \left(r^{\prime }-r^{\prime \prime }\right)\delta \left(p^{\prime }-p^{\prime \prime }\right)\frac{\partial f(r^{\prime },p^{\prime })}{\partial p_{k}^{\prime }}\frac{\partial V(|r^{\prime }-r|)}{\partial r_{k}^{\prime }}}}. \end{array}$$

The contribution to the macroscopic evolution equations is the projection of

(71) $$\begin{array}{cc} \langle \frac{\delta J(x(t))}{\delta x} & {|}_{x=\tilde{x}\left(y\right)},J\left(x\left(t\right)\right){|}_{x=\tilde{x}\left(y\right)}\rangle =\int dr\int dp\frac{\delta J(f\left(r^{\prime \prime },p^{\prime \prime }\right)}{\delta f(r,p)}J\left(f\left(r,p\right)\right){|}_{f=\tilde{f}}=\\ & =\frac{p_{j}^{\prime \prime }p_{k}^{\prime \prime }}{m^{2}}\frac{\partial^{2}\tilde{f}\left(r^{\prime \prime },p^{\prime \prime }\right)}{\partial r_{j}^{\prime \prime }\partial r_{k}^{\prime \prime }}+\frac{\partial }{\partial r_{k}^{\prime \prime }}\left({\tilde{F}}_{j}\left(r^{\prime \prime }\right)\frac{\partial \tilde{f}\left(r^{\prime \prime },p^{\prime \prime }\right)}{\partial p_{j}^{\prime \prime }}\right)\frac{p_{k}^{\prime \prime }}{m}\\ & +\frac{{\tilde{F}}_{k}\left(r^{\prime \prime }\right)}{m}\frac{\partial \tilde{f}\left(r^{\prime \prime },p^{\prime \prime }\right)}{\partial r_{k}^{\prime \prime }}+{\tilde{F}}_{k}\left(r^{\prime \prime }\right)\frac{p_{j}^{\prime \prime }}{m}\frac{\partial^{2}\tilde{f}\left(r^{\prime \prime },p^{\prime \prime }\right)}{\partial r_{j}^{\prime \prime }\partial p_{k}^{\prime \prime }}\\ & +{\tilde{F}}_{k}\left(r^{\prime \prime }\right){\tilde{F}}_{j}\left(r^{\prime \prime }\right)\frac{\partial^{2}\tilde{f}\left(r^{\prime \prime },p^{\prime \prime }\right)}{\partial p_{j}^{\prime \prime }\partial p_{k}^{\prime \prime }}. \end{array}$$

The first correction to the macroscopic evolution equation for density $\frac{\partial \rho }{\partial t}\left(t,r\right)$ follows from projection $\pi_{\rho }=\int dpm$ applied to this last relation and the particular form of quasi-equilibrium $\tilde{f}$ from (65),

(72) $$\begin{array}{cc} \frac{\partial \rho (t,r)}{\partial t} & =\frac{1}{2}\tau \langle \pi_{\rho },\langle J_{f},J\rangle {|}_{\tilde{f}}\rangle \\ & =\frac{\tau }{2}\left[\frac{1}{m}\int dpp_{j}p_{k}\frac{\partial^{2}\tilde{f}}{\partial r_{k}\partial r_{j}}+\int dpp_{k}\frac{\partial }{\partial r_{k}}\left({\tilde{F}}_{j}\frac{\partial \tilde{f}}{\partial p_{j}}\right)\right.\\ & \;\left.+\int dp{\tilde{F}}_{k}\frac{\partial \tilde{f}}{\partial r_{k}}+\int dp{\tilde{F}}_{k}p_{j}\frac{\partial^{2}\tilde{f}}{\partial r_{j}\partial p_{k}}+\int dpm{\tilde{F}}_{k}{\tilde{F}}_{j}\frac{\partial^{2}\tilde{f}}{\partial p_{j}\partial p_{k}}\right]\\ & =\frac{\tau }{2}\left[\frac{1}{m}\frac{\partial^{2}}{\partial r_{j}\partial r_{k}}\int dpp_{j}p_{k}\tilde{f}-\frac{\partial {\tilde{F}}_{k}}{\partial r_{k}}\frac{\rho }{m}-{\tilde{F}}_{k}\frac{\partial \rho /m}{\partial r_{k}}\right]\\ & =\frac{\tau }{3}\Delta \varepsilon -\frac{\tau }{2}\nabla \cdot \left(\tilde{F}\frac{\rho }{m}\right), \end{array}$$

where the first integral on the last but one line vanishes unless k=j due to symmetry of $\tilde{f}$ (an even function in $p_{i}$). Its nonzero value is 2εm/3 as can be seen from the projections. Note that Δ stands for the Laplacian.

In exactly the same manner, one can proceed in obtaining the first (irreversible) approximation of the macroscopic evolution equation for kinetic energy density. Finally, the reduced evolution equations become

(73) $$\begin{array}{ccc} \frac{\partial \rho }{\partial t} & = & \frac{\tau }{3}\Delta \varepsilon -\frac{\tau }{2}\nabla \cdot \left(\rho F^{\left(ME\right)}\right), \end{array}$$

(74) $$\begin{array}{ccc} \frac{\partial \varepsilon }{\partial t} & = & \frac{\tau }{2}\left(\frac{10}{9}\Delta \frac{\varepsilon^{2}}{\rho }+{\left(F^{\left(ME\right)}\right)}^{2}\rho -\nabla \cdot \left(\frac{5}{3}F^{\left(ME\right)}\varepsilon \right)-\frac{2}{3}F^{\left(ME\right)}\cdot \nabla \varepsilon \right), \end{array}$$

where the force in mechanical equilibrium $F^{\left(ME\right)}$ is defined as

(75) $$\begin{array}{ccc} F_{j}^{\left(ME\right)}\left(r^{\prime \prime }\right) & = & \frac{1}{m}F_{j}{|}_{f=\tilde{f}}=-\frac{1}{m}\int dr^{\prime }\int dp^{\prime }\frac{\partial V(|r^{\prime \prime }-r^{\prime }|)}{\partial r_{j}^{\prime \prime }}\tilde{f}\left(r^{\prime },p^{\prime }\right)\\ & = & -\frac{1}{m^{2}}\int dr^{\prime }\frac{\partial V(|r^{\prime \prime }-r^{\prime }|)}{\partial r_{j}^{\prime \prime }}\rho \left(r^{\prime }\right). \end{array}$$

Equations (73) and (74) are the evolution equations approximating the Vlasov equation on the level of mechanical equilibrium, and they have been derived by the Ehrenfest reduction. The equations are clearly irreversible in the sense of time-reversal transformation [34].

In summary, the reduced evolution on the level of mechanical equilibrium is irreversible although the detailed evolution (Vlasov Equation (1)) was completely reversible, and entropy on the level of mechanical equilibrium is expected to grow in time although the Boltzmann entropy is conserved by the Vlasov equation. Let us further analyze the evolution equations in the next section.

### 3.3. Features of the Reduced Evolution

#### 3.3.1. Conservation of Total Energy

Evolution of the total energy on the level of mechanical equilibrium

(76) $$E^{\left(ME\right)}\left(\rho ,\varepsilon \right)=\int dr\varepsilon +\frac{1}{2}\int dr\int dr^{\prime }\frac{\rho (r)}{m}V(|r-r^{\prime }\left|\right)\frac{\rho (r^{\prime })}{m}$$

can be obtained by using the reduced evolution Equations (73) and (74) through

(77) $$\begin{array}{ccc} {\dot{E}}^{\left(ME\right)} & = & \int dr\partial_{t}\varepsilon +\int dr\int dr^{\prime }\frac{\rho (r)}{m}V(|r-r^{\prime }\left|\right)\frac{\partial_{t}\rho \left(r^{\prime }\right)}{m}\\ & = & \int dr\frac{\tau }{2}\left({\left(F^{\left(ME\right)}\right)}^{2}\rho -\frac{2}{3}F^{\left(ME\right)}\cdot \nabla \varepsilon \right)\\ & = & +\frac{1}{m^{2}}\int dr\int dr^{\prime }\rho \left(r\right)V(|r-r^{\prime }\left|\right)\left(\frac{\tau }{3}\Delta^{\prime }\cdot \varepsilon \left(r^{\prime }\right)-\frac{\tau }{2}\nabla^{\prime }\cdot \left(\rho \left(r^{\prime }\right)F^{\left(ME\right)}\left(r^{\prime }\right)\right)\right)\\ & = & \int dr\frac{\tau }{2}\left({\left(F^{\left(ME\right)}\right)}^{2}\rho -\frac{2}{3}F^{\left(ME\right)}\cdot \nabla \varepsilon \right)\\ & = & -\frac{1}{m^{2}}\int dr\int dr^{\prime }\rho \left(r\right)\nabla^{\prime }V(|r-r^{\prime }\left|\right)\left(\frac{\tau }{3}\nabla^{\prime }\varepsilon \left(r^{\prime }\right)-\frac{\tau }{2}\cdot \left(\rho \left(r^{\prime }\right)F^{\left(ME\right)}\left(r^{\prime }\right)\right)\right)\\ & = & \int dr\frac{\tau }{2}\left({\left(F^{\left(ME\right)}\right)}^{2}\rho -\frac{2}{3}F^{\left(ME\right)}\cdot \nabla \varepsilon \right)\\ & = & -\int dr^{\prime }\frac{\tau }{2}\rho \left(r^{\prime }\right){\left(F^{\left(ME\right)}\left(r^{\prime }\right)\right)}^{2}+\int dr^{\prime }\frac{\tau }{3}F^{\left(ME\right)}\left(r^{\prime }\right)\cdot \nabla^{\prime }\varepsilon \left(r^{\prime }\right)\\ & = & 0. \end{array}$$

Total energy is thus conserved by the reduced evolution equations.

#### 3.3.2. Dissipativity of Reduced Evolution

To assess the growth of the reduced entropy, we evaluate the following expression

(78) $${\dot{S}}^{\left(ME\right)}=\int drS_{\rho }\left(r\right)\frac{\partial \rho }{\partial t}+S_{\varepsilon }\left(r\right)\frac{\partial \varepsilon }{\partial t},$$

and obtain

(79) $$\begin{array}{ccc} {\dot{S}}^{\left(ME\right)} & = & \frac{k_{B}\tau }{m}\int dr\left[\frac{3}{4}\frac{\rho^{2}}{\varepsilon }{\left(F^{\left(ME\right)}\right)}^{2}+\frac{\varepsilon }{6}\left(7{\left(\frac{\nabla \varepsilon }{\varepsilon }\right)}^{2}+5{\left(\frac{\nabla \rho }{\rho }\right)}^{2}-10\frac{\nabla \rho }{\rho }\cdot \frac{\nabla \varepsilon }{\varepsilon }\right)\right.\\ & & \left.\;\;-\frac{\rho }{\varepsilon }F^{\left(ME\right)}\cdot \nabla \varepsilon \right]. \end{array}$$

The first term on the right-hand side is always non-negative. Using the Young inequality $|a\cdot b|\leq (a^{2}+b^{2})/2$, the remaining three terms on the first line can be estimated from below as

(80) $$7{\left(\frac{\nabla \varepsilon }{\varepsilon }\right)}^{2}+5{\left(\frac{\nabla \rho }{\rho }\right)}^{2}-10\frac{\nabla \rho }{\rho }\cdot \frac{\nabla \varepsilon }{\varepsilon }\geq 2{\left(\frac{\nabla \varepsilon }{\varepsilon }\right)}^{2},$$

which means that the entropy production is bounded from below by

(81) $${\dot{S}}^{\left(ME\right)}\geq \frac{k_{B}\tau }{m}\int dr\left[\frac{3}{4}\frac{\rho^{2}}{\varepsilon }{\left(F^{\left(ME\right)}\right)}^{2}+\frac{1}{3}\frac{{\left(\nabla \varepsilon \right)}^{2}}{\varepsilon }-\frac{\rho }{\varepsilon }F^{\left(ME\right)}\cdot \nabla \varepsilon \right].$$

Unlike the first two positive terms, the last term has no definite sign, which means that it has to be estimated using the positive terms. By Hölder inequality $\left|\int drfg\right|\leq \sqrt{\int drf^{2}}\sqrt{\int drg^{2}}$ and the Young inequality, the last term can be bounded from above as

(82) $$\begin{array}{ccc} \left|\int dr\frac{\rho }{\varepsilon }F^{\left(ME\right)}\cdot \nabla \varepsilon \right| & = & \left|\int dr\frac{cF^{\left(ME\right)}\rho }{\sqrt{\varepsilon }}\cdot \frac{\nabla \varepsilon }{c\sqrt{\varepsilon }}\right|\\ & \leq & \sqrt{c^{2}\int dr{\left(F^{\left(ME\right)}\right)}^{2}\frac{\rho^{2}}{\varepsilon }}\cdot \sqrt{\frac{1}{c^{2}}\int dr\frac{{\left(\nabla \varepsilon \right)}^{2}}{\varepsilon }}\\ & \leq & \frac{1}{2}\left(c^{2}\int dr{\left(F^{\left(ME\right)}\right)}^{2}\frac{\rho^{2}}{\varepsilon }+\frac{1}{c^{2}}\int dr\frac{{\left(\nabla \varepsilon \right)}^{2}}{\varepsilon }\right), \end{array}$$

where *c* is an arbitrary constant. Setting $c^{2}/2=3/4$, i.e., $1/2c^{2}=1/3$, the last expression becomes equal to the two positive terms on the right-hand side of Equation (81), and we obtain eventually that ${\dot{S}}^{\left(ME\right)}\geq 0$.

The reduced entropy production is always non-negative, and the reduced evolution equations are thus compatible with the second law of thermodynamics.

#### 3.3.3. Homogeneous Equilibrium Solution

A natural question now arises whether there exists a (spatially) homogeneous solution of the macroscopic evolution Equations (73) and (74) describing the system in mechanical equilibrium, i.e., a solution to

(83) $$\begin{array}{cc} 0 & =\nabla \cdot F^{ME}, \end{array}$$

(84) $$\begin{array}{cc} 0 & =-\frac{5}{3}\varepsilon \nabla \cdot F^{ME}+{\left(F^{ME}\right)}^{2}\rho . \end{array}$$

Hence, one immediately arrives at a necessary condition for the existence of a homogeneous solution that states that $F^{ME}=0$, perfectly corresponding to the observation of Villani et al. that $F^{ME}$ strongly tends to zero with long times (as perturbations decay). With the knowledge of the particular form of the quasi-equilibrium $\tilde{f}$ and with the assumption of homogeneous ρ,ε we may proceed further with

$$F_{k}^{ME}=0=\frac{\partial }{\partial r_{k}}\int dr^{\prime }\int dp^{\prime }V(|r-r^{\prime }\left|\right)\tilde{f}\left(\rho ,\varepsilon ,p\right)=const\frac{\partial }{\partial r_{k}}\int dr^{\prime }V(|r-r^{\prime }\left|\right)$$

to observe that a homogeneous solution exists if

(85) $$\begin{array}{cc} \Omega =R^{3}\;\mathrm{and} & \int_{\Omega }drV\left(|r|\right)<+\infty , \end{array}$$

(86) $$\begin{array}{cc} \mathrm{or}\;\Omega \;\text{is periodic as a system} & \left(e.g.,\text{a torus}\right)\Rightarrow \frac{\partial }{\partial r_{k}}\int dr^{\prime }V(|r-r^{\prime }\left|\right)=0, \end{array}$$

revealing a nontrivial condition for the interacting potential for unbounded regions (again, cf. findings about linear and nonlinear Landau damping). The latter condition is due to the possible change of variables such that the expression under gradient does not depend on position. Fast enough decay of the potential in case of infinite domain or periodicity of the domain itself are necessary conditions for existence of a stationary homogeneous solution. In such cases, any constant is a solution, and conditions (85) and (86) are thus also sufficient.

The choice of the homogeneous solution is not, however, arbitrary. It follows from the initial condition and the evolution equation accompanied by entropy functional guiding this process. To prove mathematically that the reduced evolution equations tend to this homogeneous solution is beyond the scope of this paper (if it can be done at all: strongly coupled nonlinear parabolic PDEs). However, the approach of ρ, precise evolution of which is given by the Vlasov equation, to a homogeneous equilibrium was proven by Villani and Mouhot as a manifestation of nonlinear Landau damping. The approach of ρ to a homogeneous equilibrium by means of the reduced evolution Equations (73) and (74) can be anticipated.

Is the homogeneous solution the state where a maximum of the mechanical equilibrium entropy is reached? The system of evolution equations is accompanied by entropy functional (66) indicating the evolution of mechanical equilibrium variables ρ and ε towards the equilibrium values. Maximization of the reduced entropy with respect to constraints

(87) $$M\left(\rho ,\varepsilon \right)=\int dr\rho \left(r\right),\;E\left(\rho ,\varepsilon \right)=\int dr\varepsilon +\frac{1}{m^{2}}\frac{1}{2}\int dr\int dr^{\prime }\rho \left(r\right)V(|r-r^{\prime }\left|\right)\rho \left(r^{\prime }\right)$$

can be written as

(88) $$\begin{array}{c} \frac{\delta }{\delta \rho }\left(-S^{\left(ME\right)}+E^{*}\cdot E\left(\rho ,\varepsilon \right)+M^{*}\cdot M\left(\rho ,\varepsilon \right)\right)=0, \end{array}$$

(89) $$\begin{array}{c} \frac{\delta }{\delta \varepsilon }\left(-S^{\left(ME\right)}+E^{*}\cdot E\left(\rho ,\varepsilon \right)+M^{*}\cdot M\left(\rho ,\varepsilon \right)\right)=0. \end{array}$$

These equations lead to

(90) $$\begin{array}{ccc} E^{*} & = & \frac{3}{2}k_{B}\frac{\rho }{m\varepsilon }, \end{array}$$

(91) $$\begin{array}{ccc} M^{*} & = & -k_{B}\frac{3}{2m}\ln\frac{3h^{2}}{4\pi m^{2}}\frac{\rho }{\varepsilon }-\frac{k_{B}}{m}\ln\frac{\rho }{m}-E^{*}\frac{1}{m^{2}}\int dr^{\prime }V(|r-r^{\prime }\left|\right)\rho \left(r^{\prime }\right). \end{array}$$

Therefore, there is a relation between particle density and kinetic energy density (in the homogeneous equilibrium) via a constant α

(92) $$\varepsilon =\alpha \rho ,\;\alpha =\frac{3}{2}k_{B}T/m,\;T^{-1}=E^{*},$$

and consequently total energy becomes for homogeneous distribution of particles

(93) $$E=\alpha M+\frac{1}{2m^{2}}\nu \bar{\rho }M,$$

where ν=∫drV(|r|), $\bar{\rho }=M/\left|\Omega \right|$, $\bar{\varepsilon }=E/\left|\Omega \right|$ and Ω is the spatial integration domain. The MaxEnt problem has thus a solution homogeneous in space, and the solution has been determined explicitly (for known total energy and mass). Is the solution unique?

To proceed further and comply with the definitions of energies above, we need to restrict ourselves to finite domains. Hence, we are forced further to assume that Ω is a finite domain, e.g., a torus, in line with the assumptions in the work of Mouhot and Villani [1]. This identifies constant α as function of *E*, *M*, Ω and of the potential *V*. Moreover, Equation (91) gives the Lagrange multiplier $M^{*}$ as a function of *E*, *M* and Ω as

(94) $$M^{*}=-k_{B}\frac{3}{2m}\ln\left(\frac{3h^{2}}{4\pi m^{2}}\frac{1}{\alpha (E,N,\nu ,\Omega )}\right)-\frac{k_{B}}{m}\ln\frac{\bar{\rho }}{m}-\frac{3}{2}\frac{k_{B}}{m^{3}\alpha \left(E,N,\nu ,\Omega \right)}\nu \bar{\rho }.$$

Equation (91) thus becomes

(95) $$\ln\rho +\frac{3}{2}\frac{1}{m^{2}\alpha }\int dr^{\prime }V(|r-r^{\prime }\left|\right)\rho \left(r^{\prime }\right)=\ln\bar{\rho }+\frac{3}{2}\frac{1}{m^{2}\alpha }\nu \bar{\rho },$$

which is a nonlinear integral equation for ρ(r).

Equation (95) can be linearized around $\bar{\rho }$ through $\tilde{\rho }:=\frac{\rho }{\bar{\rho }}-1\ll 1$ in magnitude, which yields

(96) $$\tilde{\rho }+\frac{3}{2}\frac{1}{m^{2}\alpha }\bar{\rho }\int dr^{\prime }V(|r-r^{\prime }\left|\right)\tilde{\rho }\left(r^{\prime }\right)=0,$$

which is a linear Fredholm integral equation. By applying Fourier transform to the equation, we obtain that

(97) $$\hat{\rho }+\frac{3}{2}\frac{\bar{\rho }}{m^{2}\alpha }\hat{V}\hat{\rho }=0,$$

where $\hat{\rho }\left(\xi \right)=\int dr\tilde{\rho }\left(r\right)\exp\left(-2\pi ir\cdot \xi \right)$. The transformed equation means that $\hat{\rho }=0$ and thus $\tilde{\rho }$ has to be constant. From the definition of $\tilde{\rho }$, it follows that it must be zero everywhere, which means that the only solution to the linearized problem is the homogeneous in space solution.

We have shown using physical (not mathematical) tools that there is a (at least to some extent unique) homogeneous solution to the macroscopic problem (83) and (84), which is a MaxEnt equilibrium (maximizes the reduced entropy). Moreover, entropy production is non-negative until this homogeneous density distribution is reached. Therefore, this approach is providing thermodynamic arguments for linear Landau damping.

#### 3.3.4. Some Qualitative Insight into Macroscopic Evolution Equations: Linearization.

It was shown in the preceding section that homogeneous equilibrium is a state ("the" state when near to the equilibrium) for which the mechanical equilibrium entropy $S^{\left(ME\right)}$ attains its maximum. The approach of ρ and ε by means of evolution Equations (73) and (74) towards the homogeneous equilibrium can be thus expected.

We can gain some analytical insight into the governing macroscopic evolution equations from their analytical solution, which is available only under certain assumptions. We shall thus solve a linearized version of the equations around constant solutions.

The macroscopic evolution Equations (73) and (74) can posses a constant (both in time and space) solution $\bar{\epsilon },\bar{\rho }$ only when $F^{\left(ME\right)}=0=\bar{\rho }\int dr^{\prime }\frac{\partial V(|r^{\prime \prime }-r^{\prime }|)}{\partial r_{j}^{\prime \prime }}$, which requires the system Ω to be periodic as discussed above. Then, actually arbitrary constant $\bar{\epsilon },\bar{\rho }$ is a solution, but the thermodynamically admissible single one is chosen by the entropy functional as described above.

Denoting small perturbations around this constant solution with tildes, we may approximate the evolution equations to the leading (linear) order as follows: (${\tilde{\bar{F}}}^{\left(ME\right)}$ stands for ${\tilde{\bar{F_{j}}}}^{\left(ME\right)}=-\frac{1}{m^{2}}\int dr^{\prime }\frac{\partial V(|r^{\prime \prime }-r^{\prime }|)}{\partial r_{j}^{\prime \prime }}\left(\bar{\rho }\left(r^{\prime }\right)+\tilde{\rho }\left(r^{\prime }\right)\right)$

(98) $$\begin{array}{ccc} \frac{\partial \tilde{\rho }}{\partial t} & = & \frac{\tau }{2}\left[\frac{2}{3}\Delta \left(\bar{\varepsilon }+\tilde{\varepsilon }\right)-\nabla \cdot \left(\left(\bar{\rho }+\tilde{\rho }\right){\tilde{\bar{F}}}^{\left(ME\right)}\right)\right]\\ & \approx & \frac{\tau }{2}\left[\frac{2}{3}\Delta \tilde{\varepsilon }+\frac{\bar{\rho }}{m^{2}}\int dr^{\prime }\Delta V(|r-r^{\prime }\left|\right)\tilde{\rho }\left(r^{\prime }\right)\right], \end{array}$$

(99) $$\begin{array}{ccc} \frac{\partial \tilde{\varepsilon }}{\partial t} & = & \frac{\tau }{2}\left[\frac{10}{9}\Delta \left(\left(\bar{\rho }+\tilde{\rho }\right){\left(\frac{\left(\bar{\varepsilon }+\tilde{\varepsilon }\right)}{\left(\bar{\rho }+\tilde{\rho }\right)}\right)}^{2}\right)-\nabla \cdot \left(\frac{5}{3}\left(\bar{\varepsilon }+\tilde{\varepsilon }\right){\tilde{\bar{F}}}^{\left(ME\right)}\right)-\frac{2}{3}F^{\left(ME\right)}\cdot \nabla \tilde{\varepsilon }+\left(\bar{\rho }+\tilde{\rho }\right){\left({\tilde{\bar{F}}}^{\left(ME\right)}\right)}^{2}\right]\\ & \approx & \frac{\tau }{2}\left[-\frac{10}{9}{\left(\frac{\bar{\varepsilon }}{\bar{\rho }}\right)}^{2}\Delta \tilde{\rho }+\frac{20}{9}\frac{\bar{\varepsilon }}{\bar{\rho }}\Delta \tilde{\varepsilon }+\frac{5}{3}\frac{\bar{\varepsilon }}{m^{2}}\int dr^{\prime }\Delta V(|r-r^{\prime }\left|\right)\tilde{\rho }\left(r^{\prime }\right)\right]. \end{array}$$

Fourier transform in space from $\left(\tilde{\rho }\left(r\right),\tilde{\varepsilon }\left(r\right)\right)$ to $\left(\hat{\rho }\left(\xi \right),\hat{\varepsilon }\left(\xi \right)\right)$ turns the evolution equations to

(100) $$\partial_{t}\left(\begin{array}{c} \hat{\rho }\\ \hat{\varepsilon } \end{array}\right)=-\tau \pi^{2}\xi^{2}\underset{\overset{\mathrm{def}}{=}M}{\underset{︸}{\left(\begin{array}{cc} 2\frac{\bar{\rho }}{m^{2}}\hat{V} & \frac{4}{3}\\ -\frac{20}{9}\alpha^{2}+\frac{20}{6}\alpha \frac{\bar{\rho }}{m^{2}}\hat{V} & \frac{40}{9}\alpha \end{array}\right)}}\cdot \left(\begin{array}{c} \hat{\rho }\\ \hat{\varepsilon } \end{array}\right).$$

Taking a radially symmetric potential of form $V\left(|r|\right)=Kr^{-k}$, *K* being a constant, Fourier transform of the potential is

(101) $$\int drV\left(|r|\right)\exp\left(-2\pi ir\cdot \xi \right)=K\frac{\Gamma \left(\frac{-k+d}{2}\right)}{\Gamma \left(k/2\right)\pi^{-k+d/2}}{\left|\xi \right|}^{k-d},$$

*d* being the dimension. Assuming 0<k<d, the Gamma functions are always positive, which means that the Fourier transform is real and positive for K>0 (electrostatic case) while negative for K<0 (gravitational case). In the electrostatic case, the first principal minor of the matrix M is positive and determinant of the matrix,

(102) $$\det M=\frac{80}{27}\alpha^{2}+\frac{40}{9}\alpha \frac{\bar{\rho }}{m^{2}}\hat{V},$$

is also positive. The matrix is then positive definite, and the linearized system of ordinary differential Equations (100) for each Fourier mode has a solution decaying exponentially fast to zero. Therefore, the potential (or electrostatic) energy also decays exponentially. The potential energy is the $L^{1}$ norm of the convolution of the density, potential and density. By Young-Hölder inequality, it is lower than the $L^{1}$ norm of the potential multiplied by a square of the $L^{2}$ norm of density. The latter is equal to the square of the $L^{2}$ norm of the Fourier image of density, which decays exponentially in time. Therefore, potential energy also decays exponentially in time. In the gravitational case, however, the definiteness of the matrix is lost for long enough Fourier modes as in the Jeans instability.

Due to linearity of Fourier transform, the decay of perturbations occurs iff $F^{-1}\left[e^{-\tau \pi^{2}\xi^{2}\lambda_{\pm }t}\right]\left(t,r\right)$ decay with time, where we denoted $\lambda_{\pm }$ the eigenvalues of matrix M. With known potential *V*, we may readily assess this condition. To illustrate this, we further assume that the dependence of $\lambda_{\pm }$ on $\hat{V}\left(\xi \right)$ can be dropped, i.e., $\bar{\varepsilon }\gg {\bar{\rho }}^{2}\hat{V}\left(\xi \right)$, and hence $\lambda_{\pm }\xi^{2}\propto -\xi^{2}$. Thence, ε,ρ are a linear combination of two fundamental solutions to the diffusion operator. Particularly, we have

(103) $$\varepsilon \approx C_{+}\frac{1}{{\left(4\pi {\bar{\lambda }}_{+}t\tau \right)}^{3/2}}\exp\left(-\frac{r^{2}}{4{\bar{\lambda }}_{+}t\tau }\right)+C_{-}\frac{1}{{\left(4\pi {\bar{\lambda }}_{-}t\tau \right)}^{3/2}}\exp\left(-\frac{r^{2}}{4{\bar{\lambda }}_{-}t\tau }\right)$$

with ${\bar{\lambda }}_{\pm }=\frac{4\bar{\varepsilon }}{9\bar{\rho }}\left(5\pm \sqrt{10}\right)$. Therefore, the initial delta pulse (at the origin) is flattening out with time as ${\left(\kappa t\tau \right)}^{-3/2},$ which can be used for identification of the essential parameter in the Ehrenfest reduction, the time-scale τ. Experiments measuring the Landau damping report characteristic times of these decays, hence assuming exponential decay. Note, however, that $C_{1}t^{-3/2}+C_{2}$ is practically indistinguishable for exponential decay over finite time intervals and real data. Finally, note that one should convolve the initial condition with the above identified responses to delta pulses, but we argue that such a response is already providing a qualitative insight into long-time behavior (decay).

In summary, linearization of Equations (73) and (74) around homogeneous solutions leads to the conclusion that the equations approach the homogeneous solutions exponentially, which is compatible with maximization of entropy at the homogeneous solutions and Landau damping. Landau damping can thus be manifestly visible on the less detailed level of mechanical equilibrium.

## 4. Conclusions

Landau damping is the property of the distribution function, governed by reversible Vlasov equations, that approaches spatially homogeneous equilibria in a weak sense (i.e., in the mean). Particle density (integral of the distribution function) then approaches the homogeneous equilibrium strongly. The reversible Vlasov equation does not alter the Boltzmann entropy, which depends on the distribution function. Conservation of entropy and the approach to the homogeneous equilibrium (Landau damping) are thus seemingly in contrast.

Here, we have shown (by means of the Ehrenfest reduction) that the Vlasov equation can be approximated by evolution equations for particle density ρ and kinetic energy density ε, (73) and (74). These state variables are equipped with their own entropy, (66), which is given by maximization of the Boltzmann entropy. The evolution equations approach homogeneous equilibrium, where the reduced entropy is maximized. It is thus the reduced hydrodynamic entropy (66) that is maximized during the Landau damping, and Landau damping has been given an alternative thermodynamic interpretation. The paradox of thermodynamics of Landau damping is resolved this way.
